# Partial Reprogramming Is Conserved from Insect to Mammal

**DOI:** 10.3390/cells15020168

**Published:** 2026-01-16

**Authors:** Nicholas S. Tolwinski, Sheng Fong, Sujithra Shankar, Jan Gruber

**Affiliations:** 1 Program in Cancer and Stem Cell Biology, Duke-NUS Medical School, Singapore 169857, Singapore; sujithra.shankar@duke-nus.edu.sg; 2 Population Health Research Office, Ng Teng Fong General Hospital, Singapore 609606, Singapore; fong_sheng@nuhs.edu.sg; 3 Department of Medicine (Geriatric Medicine), Ng Teng Fong General Hospital, Singapore 609606, Singapore; 4 Department of Biochemistry, Yong Loo Lin School of Medicine, National University of Singapore, Singapore 117596, Singapore; jangruber467@gmail.com; 5 Healthy Longevity Translational Research Program, Yong Loo Lin School of Medicine, National University of Singapore, Singapore 117456, Singapore

**Keywords:** reprogramming, Senotherapeutic, OKSM, OSKM, aging, longevity

## Abstract

As we become older, systems throughout the body gradually decline in function. Contributing factors include the accumulation of senescent cells and the dysfunction and exhaustion of stem and progenitor cells. A promising approach to mitigate these changes and enhance cellular function in aged animals is the discovery that differentiated cells retain plasticity, enabling them to revert to pluripotent states when exposed to Yamanaka factors. This method has shown promise in models of rapid aging, and recent studies have demonstrated notable life extension in both flies and mice. These findings, along with the development of senolytics and aging clocks, could revolutionize aging research and interventions. Here, we review recent discoveries in the field and propose new directions for intervention discovery.

## 1. Introduction

A fundamental question in developmental biology has been whether cells, once specialized, can revert to a less specialized state and adopt a different fate, or if only germ cells possess this ability. Foundational experiments with nuclear transplantation in frogs [1,2,3] and the derivation of pluripotent embryonic stem cell lines in mice [4,5] and humans [6] proved that cellular potency can be reset. A breakthrough came with the discovery that expression of four transcription factors could reprogram differentiated cells back to a pluripotent state [7,8]. Cells once thought to be terminally differentiated can be induced into a state resembling that of embryonic stem cells. These cells are called induced pluripotent stem (iPS) cells. They are stem cells derived from differentiated adult cells, such as skin or blood cells, that have been reprogrammed into an embryonic-like state. iPS cells can differentiate into many cell types, offering potential therapies for replacing lost cells in the body. They can be generated from patient-specific cells, avoiding the need for immune suppression to prevent rejection in regenerative treatments.

In the context of aging, cellular reprogramming can reverse key molecular signatures of age. During full reprogramming to pluripotency, age-related epigenetic features (such as DNA methylation and chromatin modifications) are largely reset [9]. Conversely, partial reprogramming temporarily activates reprogramming factors to rejuvenate parts of the epigenome and transcriptome without eliminating somatic identity, shifting cells toward a younger molecular state [10,11]. Notably, the resulting states are not identical to embryonic or youthful somatic cells: iPSCs may retain epigenetic memory and exhibit aberrant reprogramming hotspots, and partial reprogramming produces a rejuvenated yet distinct somatic state rather than full pluripotency [10,12]. Significantly, *“rejuvenation” in this context is inferred from aging clock predictions during reprogramming, making it a fragile concept, since most clocks face challenges with data outside their typical range* [13]. Furthermore, both theoretical and empirical evidence suggest that inherently entropic or irreversible aspects of aging (such as certain types of macromolecular damage, DNA or mtDNA mutations) might not be completely reset [14].

These considerations motivate cell rejuvenation through partial reprogramming as a practical strategy to restore tissue function. As we age, tissue-maintaining stem-cell pools decline due to exhaustion and loss, which affects their ability to divide properly (including asymmetric and symmetric divisions), thus impairing homeostasis [15,16,17]. Partial reprogramming could reset stem cells to a younger state and restore their division dynamics to repopulate niches, supporting repair and maintenance. Historically, approaches involved ex vivo stem-cell transplantation; in vivo expression of Yamanaka factors aims to stimulate endogenous cells, potentially improving safety and scalability by eliminating the need to grow cells ex vivo and transplant them. In this approach, the body’s own cells are stimulated to counteract the effects of aging and age-related diseases.

Expression scopes differ by model, with Yamanaka factors expressed systemically in some studies and cell-type specifically in others. While pan-tissue rejuvenation is an appealing idea, current evidence indicates effects depend on the context. In cases of neurodegeneration, partial reprogramming may restore cells with defective responses to misfolded proteins, leading to improved proteostasis and cellular resilience [18,19,20,21]. Overall, when properly dosed and targeted, partial reprogramming can improve homeostasis in aged organisms, but its cellular mediators, limitations, and broader applicability need further investigation. Identifying which cells contribute to rejuvenation and health span benefits requires additional research. For example, the Yamanaka factors could be tested in a post-mitotic organism like *C. elegans* to determine if the effects persist in an organism lacking stem cells besides the germline. A recent preprint using worm orthologs showed heterogeneous effects on lifespan [22]. Regardless of which cells are impacted, the treatment seems to restore homeostasis in aged individuals and prevent age-related diseases.

Mouse and in vitro models for cell rejuvenation have been used to test various tissues for improvements in biological age. Single-dose [23], multi-dose [24], liver plasticity [25], retinal regeneration [26], stem cells [27], and many other experiments have been conducted. The Hutchinson–Gilford Progeria mouse model [28] was recently followed by a genomic-damage model that showed both forward and backward shifts in biological age, highlighting the inherent plasticity of the aging process [29]. Importantly, early efforts to translate cellular rejuvenation into humans are already underway. Several studies are exploring how to apply this technology to human aging and age-related diseases; for example, rejuvenation of the retina can restore vision and de-age various human cells in vitro [11,26,27,30,31,32,33,34,35].

**Figure 1:**
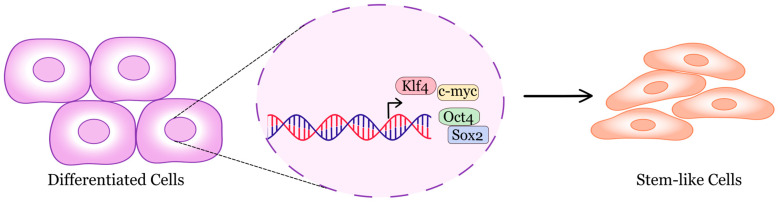
Schematic representation of reprogramming with Yamanaka factors.

In early research, induced pluripotent stem cells (iPSCs) were generated from fibroblasts obtained from patients with Hutchinson–Gilford progeria syndrome, a rare and deadly premature-aging disease. The iPSCs lacked the nuclear envelope abnormalities and epigenetic changes associated with early aging, and when differentiated into smooth muscle cells, they displayed premature senescence phenotypes linked to vascular aging [30]. Later, it was reported that it is possible to reprogram adult cells into iPS cells within tissues in vivo. The authors showed that inducing Oct4, Sox2, Klf4, and c-Myc in mice led to the formation of teratomas from multiple organs, indicating full reprogramming in vivo [31] (Figure 1). A major advancement was the in vivo doxycycline-inducible expression of reprogramming factors. They found that even the brief expression of these factors in various tissues resulted in tumors composed of undifferentiated dysplastic cells [32].

Tumor formation in these studies was disappointing from the perspective of preventing age-related decline, but they did show some tissue improvements. Suspecting that dosing was a key factor, the next study found that partial reprogramming via short-term expression of the Yamanaka factors enhanced cellular and physiological markers of aging. It extended the lifespan of a mouse model of premature aging without tumor formation, and in older wild-type mice, it improved recovery from metabolic disease and muscle injury [36].

After resolving the dosing issue, further studies focused on rejuvenating effects across various tissues and at the organism level. This research demonstrated that the length of treatment impacted the degree of beneficial effects, with longer-term partial reprogramming being more effective at delaying aging phenotypes than short-term reprogramming. The rejuvenating effects were associated with a reversal of the epigenetic clock and alterations in metabolism and gene expression, including reductions in genes linked to inflammation, senescence, and stress response pathways [24].

Another approach involved studying the role of epigenetic changes in aging and how they affect tissue function and regenerative capacity [37]. Using the mouse eye as a model to explore how aging impacts the central nervous system, researchers found that ectopic expression of the genes Oct4, Sox2, and Klf4, excluding c-Myc, in mouse retinal ganglion cells restored youthful DNA methylation patterns and transcriptomes. This led to improved axon regeneration and reversed vision loss in a mouse model of glaucoma and in aged mice. The study showed that youthful epigenetic information, partly encoded by DNA methylation, persists in older tissues and can be accessed to enhance tissue function and support regeneration in vivo [9].

The eye was not the only organ affected. Another study focused on adult cardiomyocytes, using all four factors to reactivate regenerative capacity in adult hearts. Short-term expression of OSKM before and during myocardial infarction improved cardiac function and reduced myocardial damage, suggesting that temporary dedifferentiation and reprogramming could aid heart regeneration [38].

Reprogramming shows functional benefits in different organs. In skeletal muscle, cyclic OSKM enhances regeneration after injury and reduces fibrosis [36,39]. Interestingly, it is not just about reprogramming satellite stem cells; when OSKM is expressed in myofibers, those fibers alter their secretory profile (e.g., lowering Wnt4), which then activates the stem cell niche in a paracrine manner [30,40]. Non-viral plasmid delivery and even transient mRNA pulses into muscle stem cells cause increased proliferation, restored “stemness” in aged cells, and enhanced force production after transplantation—showing that different delivery methods can cause the same temporary rejuvenation.

The brain and visual system offer some of the most remarkable examples. In aged or injured scenarios, partial reprogramming encourages astrocyte and neuronal progenitor growth, expands neuroblast pools, and improves the survival of new neurons [41]. In models where expression is confined to neurons and cycled over months, animals exhibit better memory and cognitive abilities, reduced immune-reactive extracellular matrix, and, in neurodegeneration models, fewer amyloid plaques and stronger synaptic integrity [21,42]. In the optic nerve and retina, delivering OSK (excluding c-Myc) supports retinal ganglion cell survival, promotes axon regrowth, and restores aspects of vision—effects connected to epigenetic remodeling involving TET enzymes. These neural studies emphasize a key theme: it is possible to trigger a protective, restorative state without reverting cells to a pluripotent state [43,44].

Regenerative benefits extend into tissues where fibrosis and scarring limit function. In the skin, partial reprogramming speeds up wound healing and decreases scar formation; the dermis recovers more like youthful tissue, with a better-organized extracellular matrix [24,45]. In the heart, brief OSKM exposure around ischemic injury encourages cardiomyocytes to enter a fetal-like, proliferative state, decreases scar size, and enhances cardiac performance—showing that even mostly post-mitotic cells can temporarily loosen their constraints to improve repair [38].

Epithelial organs that naturally adapt respond vigorously. In the liver, partial reprogramming promotes hepatocyte growth and survival despite toxins, accompanied by dedifferentiation into fetal-like states similar to those seen after severe injury [25,30]. Mechanistically, this reflects natural regeneration pathways: pioneer factors in the SOX family (such as SOX4) can open new chromatin regions and displace lineage-defining factors like HNF4α from their enhancers, temporarily shutting down the hepatocyte identity program to enable repair [11,27]. In the intestine, transient OSKM expression produces fetal-like epithelial cell populations and speeds up regeneration even without clear injury [46].

Other organs offer complementary benefits. In models using alveolar type II cells, partial reprogramming enhances progenitor traits and clonogenic ability, and when these cells are transplanted into injured lungs, they reduce fibrosis and improve function [47]. In the pancreas, brief OSKM induction during recovery from chemical injury improves glucose tolerance and increases beta cell mass, indicating that temporary dedifferentiation and proliferative bursts can restore endocrine function.

Possible mechanisms likely include transcription factor binding and chromatin accessibility [48,49]. At high levels, OSK binds not only to their high-affinity sites but also to low-affinity composite Oct-Sox motifs scattered near previously closed regions [49,50,51,52]. This widespread opening incidentally exposes motifs for other factors—both lineage-defining and stress-responsive proteins, such as the AP-1 complex—which, with age and chronic stress, increasingly occupy stress-response chromatin and act as pioneer factors, keeping those regions open [53,54,55,56]. Partial reprogramming’s global chromatin opening may temporarily redistribute AP1 and other cofactors, allowing the stress loci associated with aging to close when OSKM is withdrawn. The overall effect is a rebalancing: identity loci regain occupancy, stress-response accessibility decreases, and cells recover a more youthful transcriptional profile. This links the erasure of aging “stress memory” with the regenerative improvements seen in multiple tissues.

To examine the mechanism of partial reprogramming, one study used single-cell genomics to map changes caused by partial pluripotent reprogramming in mouse cells and to understand the role of individual Yamanaka Factors (Oct4, Sox2, Klf4, and c-Myc) in this process. The results showed that partial reprogramming could restore youthful gene expression in adipogenic and mesenchymal stem cells but also temporarily suppressed their somatic identity. The study also found that different combinations of Yamanaka Factors could have varying effects on restoring youthful expression and suppressing somatic identity, indicating that different combinations of reprogramming factors can influence the outcome of pluripotent reprogramming differently [27].

In human cells, transient expression of Yamanaka factors, delivered through mRNA, can prevent cellular aging by resetting the epigenetic clock, reducing inflammation in chondrocytes, and restoring the youthful regenerative capacity of aged muscle stem cells while preserving cellular identity. These findings indicate that transient reprogramming can rejuvenate human cells and counteract age-related functional decline [30].

A technical advancement called “maturation phase transient reprogramming” (MPTR) was developed to rejuvenate various cellular traits in middle-aged dermal fibroblasts. MPTR involves selectively expressing reprogramming factors until a rejuvenation point, then removing them. This method rejuvenates the transcriptome, epigenome, and cellular features such as collagen protein production and migration speed. The rejuvenation achieved by MPTR indicates that it is possible to separate rejuvenation from full pluripotency reprogramming [11].

## 2. Lifespan Extension

The four Yamanaka factors (Oct4, Klf4, Sox2, c-Myc) include the well-known oncogene c-Myc [57,58]. Expression of these factors in mice caused teratoma formation and did not yield positive effects on aging or age-related diseases [59,60]. This issue was addressed by using repeated, short-term expression in adult mice [28,36,61]. This approach allows partial reprogramming without causing teratomas, leading to cellular rejuvenation and reducing cellular and physiological signs of aging in a premature aging, Hutchinson–Gilford Progeria mouse model [28,62,63]. A recent study suggests that removing c-Myc could be the way forward, but a direct comparison has not yet been conducted [26,29,64].

**Figure 2:**
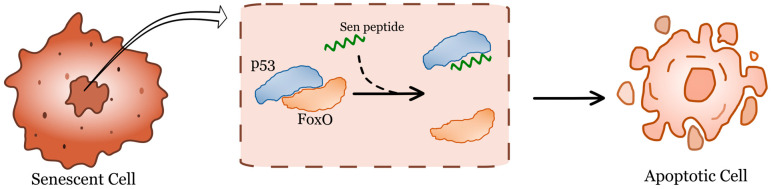
Schematic of FoxO-like senolytic peptide that interferes with FoxO binding to p53.

All these studies showed improvements in multiple hallmarks of aging, yet none assessed whether these interventions ultimately impacted lifespan. This gap was recently addressed in an invertebrate aging model, *Drosophila*, where pulsed expression of Yamanaka factors led to a significant extension in lifespan [65]. Although the study did not demonstrate a substantial improvement in healthspan with Yamanaka factors alone, the addition of a senolytic peptide notably compressed the mortality curve. This underscores the advantage of fast-aging models, as multiple interventions can be combined to investigate synergistic effects. The study suggests that combining rejuvenation strategies with secondary treatments is likely the most effective approach. Senolytics have been identified as highly promising for eliminating senescent cells, reducing chronic inflammation, and preventing tissue damage [66] (Figure 2). Combination therapy with senolytics and rejuvenation showed synergy. The idea is that removing senescent cells, which contribute to aging and age-related diseases, and replenishing the body with healthy, functioning stem cells could promote the rejuvenation of damaged or aging tissues and organs. The goal of this combination therapy is to improve overall health, delay the onset of age-related diseases, and enhance the effectiveness of other treatments.

More recently, a mouse study confirmed that these lifespan-extending benefits are conserved in mammals. Aged mice with pulsed expression of OSK, excluding c-Myc, showed a significant increase in lifespan [64]. This study, combined with human cell experiments [11,67] and findings from the evolutionarily distant *Drosophila*, suggests that this cellular property is conserved and may prove effective in humans as well.

Importantly, dosing appears to be crucial. A temporary (timed) expression of OSK can achieve significant anti-aging effects in mice. Expression of OSKM in flies also yielded different outcomes when the dosing was adjusted [65]. Precise calibration of expression levels, timing, and other factors will be needed in future studies, but the results so far indicate potential for these treatments.

## 3. Drosophila as a Model System for Reprogramming

*Drosophila* provides a powerful, genetically tractable in vivo model for the investigation of a variety of human-related processes, including reprogramming, senescence-like states, and their interplay with stress and regeneration [68,69]. Although the fly does not recapitulate mammalian pluripotency or telomere-driven replicative senescence, many core pathways governing cell-cycle exit, DNA damage responses, chromatin remodeling, inflammatory signaling, and growth control are deeply conserved [70,71,72,73]. This makes *Drosophila* an informative alternative model for systematically dissecting how reprogramming factors perturb differentiated tissues, how senescence-like programs emerge, and how these processes influence neighboring cells and tissue repair.

In flies, there is no single, universally accepted marker that definitively identifies senescent cells. SA β-gal alone is insufficient. Instead, senescence or senescence-like states are inferred from converging lines of evidence that mirror mammalian hallmarks. Stable cell-cycle exit is central: loss of S-phase entry as measured by BrdU/EdU incorporation, reduced mitotic markers such as phospho-Histone H3 and Cyclin B, and enforced G1 through upregulation of Dacapo (the p21/p27-like CDK inhibitor), repression of Cyclin E/E2F targets, and Fly-FUCCI reporters stuck in G1/G2 [71]. Irreversibility—failure to re-enter the cycle after withdrawing a stressor or supplying mitogens—strengthens the case. DNA damage and p53 pathway activation are typically assessed by γH2Av foci and p53 reporter activation; because flies maintain telomeres via retrotransposons rather than telomerase, damage looks different [69]. Chromatin and nuclear changes frequently accompany arrest, including increased heterochromatin marks such as H3K9me3, accumulation of HP1a, DAPI-dense foci in some contexts, alterations in Lamin levels or distribution, and nuclear enlargement [73].

A prominent feature of fly senescence-like programs is a SASP-like secretory/inflammatory output. JNK/AP-1 activation is often observed (puckered reporters, TRE-GFP), together with upregulation of matrix metalloproteinase 1 (Mmp1), induction of JAK/STAT cytokines (Unpaired/Upd ligands) and STAT activation in neighboring cells, and context-dependent outputs from TNF/Eiger, PDGF/VEGF-like Pvf ligands, and the NF-κB/Relish axis [69,74,75]. These signals produce non–cell autonomous consequences—neighboring proliferation, dedifferentiation, invasion, and immune (hemocyte) recruitment—that parallel the functional impact of the mammalian SASP, even though the specific ligands differ. Metabolic and mitochondrial stress is likewise conserved: elevated ROS, reduced mitochondrial membrane potential, altered morphology, AMPK activation, and FOXO nuclear localization, often with reduced ATP and indicators of metabolic reprogramming. Morphological shifts, such as increased cell and nuclear size and flattened epithelial architecture, and endoreplication/polyploidy are frequently observed; stable polyploidization accompanying arrest is a practical senescence-like readout in flies. Functional assays—persistence after stress removal, durable growth arrest by lineage tracing or transplantation, hemocyte recruitment and immune-mediated clearance, and genetic epistasis showing that suppressing JNK/JAK-STAT/Dacapo weakens arrest while augmenting DDR/JNK strengthens it—provide high-confidence evidence. These hallmarks tend to co-occur in common fly contexts, including oncogene-induced senescence-like states in imaginal discs driven by Ras activation or polarity stress, age-associated arrest in long-lived tissues such as fat body, muscle, and glia, and wound-edge programs that transiently engage senescence-like features to modulate regeneration and immune responses. Because many individual markers (for example, JNK activation, ROS, or autophagy changes) are not specific on their own, the most persuasive evidence in flies combines multiple independent hallmarks: stable arrest, DDR/chromatin changes, SASP-like signaling, and non–cell autonomous effects [71].

Comparative analyses reveal that many core regulatory axes are conserved between flies and mammals—p53-mediated DNA damage signaling, Rb/E2F cell cycle control, JNK/JAK-STAT/NF-κB-like inflammatory programs, Hippo-YAP/TAZ (Yorkie in flies), Myc, Polycomb/Trithorax, SWI/SNF chromatin remodeling, and heterochromatin dynamics, but some differences remain. Fly DNA methylation differs from that of mammals [76], and flies lack a p16INK4a homolog. They rely on Dacapo and Rbf for arrest. Telomeres are retrotransposon-based, so telomere attrition does not typically drive senescence [77]. Reprogramming in fly cell lines has shown some positive results, but a mammalian-style pluripotent state from OSKM-induced iPSC reprogramming has not been demonstrated [78]. Reprogramming in flies usually involves injury- or oncogene-triggered dedifferentiation or transdetermination in somatic tissues. Epigenetically, both mammals and flies exhibit SAHF-like compaction, heterochromatin remodeling, redistribution of Polycomb/Trithorax marks, and nuclear lamina alterations [79].

To test the conservation and functional consequences of mammalian reprogramming factors in a live organism, human OSKM (Oct4, Sox2, Klf4, c-Myc) was expressed in *Drosophila* using GAL4/UAS for precise control [65]. This strategy probes whether these factors act as pioneer/lineage-erasing agents in differentiated epithelia, how they intersect with stress and senescence pathways, and what their in vivo consequences are for tissue homeostasis, regeneration, and tumor-like growth. Future studies could be used to disentangle factor-specific effects—Myc’s strong growth and oncogenic activity versus Oct4/Sox2/Klf4′s lineage destabilization. Chromatin and enhancer logic could be assayed via ATAC-seq and ChIP to determine whether OSKM open stress/regeneration enhancers, displace Polycomb, or rewire transcriptional networks. Using the exact mammalian proteins enables direct comparison to mammalian datasets; this is especially valuable because flies lack a true Oct4 ortholog and have only an approximate Klf4 ortholog (*luna,* Flybase.org). Among homologs, dMyc is strongly conserved with Max and Mnt/Mxd partners; Sox2-like functions are best modeled by the SoxB1 factors Dichaete and SoxNeuro, which can exhibit pioneer-like behavior in neural contexts; and POU domain proteins such as Pdm1/Nubbin and Pdm2 share the domain architecture of Oct4 but do not reproduce its pluripotency program [80]. Expressing mammalian OSKM therefore provides functional specificity that native fly factors cannot fully replicate, especially for Oct4 and Klf4, as recently shown by the finding that *C. elegans* orthologs do not replicate OSKM effects on worm lifespan [22].

One practical concern in interpreting reprogramming and senescence data is genetic background. Uncontrolled genetic background can confound phenotypes by modulating stress sensitivity, immune responses, chromatin states, and baseline proliferation. In the study [65], isogenic lines were used, which substantially mitigates this by minimizing segregating variation.

Finally, the question of senolytic peptides in flies remains open. To date, there is no evidence that a “senolytic” selectively ablates senescent cells in *Drosophila* in vivo. Senolytic peptides—such as those disrupting FOXO4–p53 interactions—were developed and validated in mammalian systems [66]. Convincing evidence of senolysis in *Drosophila* would require demonstrating specificity (apoptosis in senescence-marked cells but not in matched non-senescent cells), a robust composite definition of senescence beyond SA β-gal, selective depletion of the marked population by lineage tracing or reporter quantification, and functional rescue of tissue performance with reduced SASP-like signaling and without general toxicity. Together, these considerations position *Drosophila* as a great model for studying reprogramming and senescence-like programs in vivo, with strong pathway-level conservation, clear practical advantages for genetic dissection, and important species-specific differences that must be respected when extrapolating from mammalian frameworks.

## 4. Chemical Approaches

There are generally two main methods for cellular reprogramming: using cell-intrinsic components such as oocyte cytoplasm and transcription factors, and applying chemical stimulation with small molecules. Some consider the latter method simpler and easier to control. Chemical reprogramming involves using chemical compounds to induce either partial or full reprogramming, similar to the process carried out by Yamanaka factors. This method has successfully converted mouse and human somatic cells into iPS-like cells using drugs such as the methylation blockers 5-azacytidine and tranylcypromine, as well as the Jun Kinase inhibitor JNKIN8 along with many others [81,82,83]. An important feature of this chemical reprogramming approach is that it does not depend on introducing exogenous “master genes” to induce pluripotency [84].

For aging, converting somatic cells into iPS cells is not required. Instead, a drug cocktail can rejuvenate cells primarily by modifying epigenetic marks. Eukaryotic aging involves the loss of epigenetic information, such as changes in DNA methylation patterns and gene expression profiles. To reverse aging, it is necessary to restore youthful DNA methylation patterns, transcript profiles, and tissue function. This process is performed without erasing the cellular identity. It suggests the potential to develop therapeutic interventions to combat aging and age-related diseases using small molecules or other chemical compounds [85].

## 5. Senotherapeutics

Although senescence is not directly relevant to partial reprogramming, senescent cells can interfere with organ regeneration perhaps even preventing some of the benefits. Cellular senescence is a fundamental mechanism underlying many age-related diseases, including metabolic dysfunction, cardiovascular disease, chronic inflammation, neurodegeneration, musculoskeletal decline, and cancer [86,87]. In response to stress and damage, senescent cells permanently exit the cell cycle but remain metabolically active, often acquiring a senescence-associated secretory phenotype (SASP) that propagates chronic inflammation and tissue dysfunction [88]. These cells accumulate with age and concentrate at sites of pathology, making them an attractive therapeutic target for managing chronic, multifactorial conditions [89].

Senotherapeutics include two main approaches: senolytics, which specifically target and eliminate senescent cells, and senomorphics, which suppress SASP. Several senolytic agents, such as dasatinib, quercetin, fisetin, and navitoclax, have been identified through hypothesis-driven screening and have demonstrated wide-ranging preclinical benefits [90]. In various mouse models, senolytics enhance physical function, slow down age-related tissue decline, reduce markers of senescence, and can extend median lifespan while compressing late-life mortality [91,92]. For instance, dasatinib combined with quercetin (D+Q) reduces intervertebral disc degeneration, maintains matrix integrity, and lowers senescence in aged mice [93]; newer agents like PZ15227 similarly rejuvenate tissue stem and progenitor cells in aged mice without causing severe thrombocytopenia [94].

These findings have positioned senolytics as a promising class of interventions for extending healthy lifespan. Yet, translation to humans requires careful evaluation of safety, dosing, and biomarkers, and alignment across animal and human studies.

Among senolytics, D+Q is the most clinically advanced, having been tested in several early-phase human studies. These trials collectively show that intermittent dosing is feasible, generally well-tolerated, and capable of modifying senescence-related biology, although definitive clinical efficacy has not yet been proven. The first-in-human study in idiopathic pulmonary fibrosis enrolled 14 participants and demonstrated good tolerability, along with short-term improvements in physical performance, despite no measurable changes in lung function [95,96]. A complementary Phase I study with nine subjects with diabetic kidney disease provided the first direct evidence of target engagement in humans, demonstrating reductions in p16^INK4a^ and p21^CIP1+^ senescent cells in adipose and skin tissue, as well as decreases in circulating SASP factors, following D+Q treatment [97].

Building on these findings, a Phase I trial in individuals with mild Alzheimer’s dementia showed that dasatinib was detectable in the cerebrospinal fluid (CSF) of four out of five participants, confirming central nervous system penetration, although quercetin remained undetectable [98]. Treatment was well tolerated and was accompanied by modulation of inflammatory and senescence-linked CSF biomarkers, although secondary cognitive and neuroimaging outcomes remained unchanged from baseline [98]. More recently, a study of 12 older adults with mild cognitive impairment and slow gait again demonstrated feasibility and safety, with reductions in circulating TNF-α; however, cognitive gains on the Montreal Cognitive Assessment were modest and not clinically meaningful, and no significant improvements in mobility or physical performance were observed [99].

Expanding this research to a larger group, a Phase II trial involving 60 postmenopausal women with osteoporosis showed no effect on the primary outcome of bone resorption. However, early increases in bone-formation markers were observed, especially among participants with higher baseline senescent-cell burden, indicating a potentially biology-dependent response to senolytic therapy [100].

Although these studies demonstrate that senolytic therapy is biologically active in humans and can engage relevant molecular pathways, most have not demonstrated clear improvements in functional aging outcomes such as cognition or physical performance. Importantly, these trials are small, open-label or early-phase, and were not designed to assess clinical effectiveness. Overall, current evidence supports the mechanistic plausibility, safety, and translational potential of senolytics like D+Q, but well-powered, placebo-controlled trials are needed to determine if targeted clearance of senescent cells can meaningfully improve human healthspan and lifespan. If Senotherapeutics prove successful in the clinic, perhaps they could be paired with future reprogramming therapies to rejuvenate organs.

## 6. Stem Cells and Age-Related Disease

Stem cell rejuvenation has been proposed as a possible treatment for Alzheimer’s disease (AD). In Alzheimer’s, there is a decline in the number and function of neural stem cells, leading to reduced neurogenesis and impaired brain function. Stem cell rejuvenation techniques aim to boost both the quantity and activity of neural stem cells to promote neurogenesis and improve brain health. This could be achieved through various methods, such as using small molecules to activate pathways involved in stem cell renewal, transplanting stem cells directly into the brain, or employing gene therapy to increase stem cell numbers [20,101]. However, these strategies remain theoretical, and delivering stem cells directly into the brain where they are needed could be challenging.

Since many age-related diseases coincide with a loss of homeostasis in organ systems, rejuvenating aged or damaged cells through stem cell therapy could have significant effects on these conditions. Activation of the pro-stem cell pathway Wnt and inactivation of the ERK and mTOR signals have already demonstrated benefits in model organisms [102,103,104,105].

## 7. A Unifying Theme?

A promising theory describes aging as a gradual shift away from an epithelial cell fate toward a mesenchymal state [67]. This process, known as mesenchymal drift (MD), is based on analysis of numerous aged human epithelial tissue samples [67]. The theory suggests that cellular aging in epithelial and other cell types results from this drift away from their original identities. It also highlights potential treatments that can restore cell fates and reverse drift, such as partial reprogramming with Yamanaka factors, which appears to reverse MD [67]. Since major hallmarks of aging appear to be conserved across different cell types [106], a unifying approach for analyzing existing datasets and testing intervention efficacy is needed. The mesenchymal drift theory provides this framework, at least for epithelial cells, and possibly for other cell types as well. This, in combination with novel age-related atlases of DNA modifications [107] and proteins [108] promises advances in the near future [109,110].

## 8. Conclusions

The promise of reprogramming as a treatment remains far off. Current approaches, such as caloric or methionine restriction [111] or reduction in clinical risk factors [112], remain the most obvious approaches for health and lifespan extension. So, although partial reprogramming continues to yield promising results, a few questions remain [113]. Do these benefits primarily come from stem cell rejuvenation, or do they also influence differentiated cells more broadly? Determining whether the effect persists in a post-mitotic organism like *C. elegans* would provide crucial mechanistic insight, clarifying whether cell division is necessary for rejuvenation and helping distinguish stem-cell–centric effects from pan-cellular ones. From a translational perspective, adenoviral delivery in mice has proven feasible and offers strong control of expression, but assessing alternative methods—especially mRNA-based delivery—could improve safety, tunability, and repeatability, while reducing risks associated with insertion and immune responses. Thorough comparisons of biodistribution, durability, off-target effects, and immune responses will be essential [114,115]. A systematic exploration of combination strategies is also warranted. Our discovery that a senolytic peptide can enhance OSKM highlights the potential for synergy, and targeting other aging-related pathways—such as mitochondrial function, nutrient sensing (AMPK/mTOR), proteostasis and autophagy, chronic inflammation and SASP, genomic stability and DNA repair, and redox balance—might further improve efficacy and extend longevity [116]. Prioritizing combinations guided by biomarkers of epigenetic age and functional metrics [112], along with careful dosing and timing to preserve cell identity, will be critical for translating partial reprogramming into safe and effective therapies.

## Data Availability

No new data were created or analyzed in this study.
